# miR-504 modulates the stemness and mesenchymal transition of glioma stem cells and their interaction with microglia via delivery by extracellular vesicles

**DOI:** 10.1038/s41419-020-03088-3

**Published:** 2020-10-22

**Authors:** Ariel Bier, Xin Hong, Simona Cazacu, Hodaya Goldstein, Daniel Rand, Cunli Xiang, Wei Jiang, Hiba Waldman Ben-Asher, Moshe Attia, Aharon Brodie, Ruicong She, Laila M. Poisson, Chaya Brodie

**Affiliations:** 1 The Mina and Everard Goodman Faculty of Life Sciences, Bar-Ilan University, Ramat-Gan, Israel; 2 Davidson Laboratory of Cell Signaling and Tumorigenesis, Hermelin Brain Tumor Center, Department of Neurosurgery, Henry Ford Hospital, Detroit, MI USA; 3 Department of Neurosurgery, Sheba Medical Center, Henry Ford Hospital, Detroit, MI USA; 4 Department of Public Health Sciences, Henry Ford Hospital, Detroit, MI USA

**Keywords:** Cancer, CNS cancer

## Abstract

Glioblastoma (GBM) is a highly aggressive tumor with poor prognosis. A small subpopulation of glioma stem cells (GSCs) has been implicated in radiation resistance and tumor recurrence. In this study we analyzed the expression of miRNAs associated with the functions of GSCs using miRNA microarray analysis of these cells compared with human neural stem cells. These analyses identified gene clusters associated with glioma cell invasiveness, axonal guidance, and TGF-β signaling. miR-504 was significantly downregulated in GSCs compared with NSCs, its expression was lower in GBM compared with normal brain specimens and further decreased in the mesenchymal glioma subtype. Overexpression of miR-504 in GSCs inhibited their self-renewal, migration and the expression of mesenchymal markers. The inhibitory effect of miR-504 was mediated by targeting Grb10 expression which acts as an oncogene in GSCs and GBM. Overexpression of exogenous miR-504 resulted also in its delivery to cocultured microglia by GSC-secreted extracellular vesicles (EVs) and in the abrogation of the GSC-induced polarization of microglia to M2 subtype. Finally, miR-504 overexpression prolonged the survival of mice harboring GSC-derived xenografts and decreased tumor growth. In summary, we identified miRNAs and potential target networks that play a role in the stemness and mesenchymal transition of GSCs and the miR-504/Grb10 pathway as an important regulator of this process. Overexpression of miR-504 exerted antitumor effects in GSCs as well as bystander effects on the polarization of microglia via delivery by EVs.

## Introduction

Glioblastomas (GBMs) are the most common and aggressive of the astrocytic tumors. They are characterized by increased proliferation and angiogenesis, invasion into the surrounding normal tissue and resistance to therapies^1^. The prognosis of patients with GBM remains extremely poor, and the median survival of GBM patients has remained around 14–16 months over the past decades^2^. Gene expression profiling studies identified GBM subtypes that are classified based on their transcriptional signatures into various molecular groups, including proneural, classical, and mesenchymal^3,4^. Recently, the profiling of DNA methylation patterns in glioma has refined these categories^5^, aligning them with the WHO 2016 diagnostic schema for glioma. These subtypes have distinct differential genetic alterations, molecular signatures, cellular phenotypes, and patient prognosis^5–7^.

GBMs contain a small subpopulation of cancer stem cells (i.e., glioma stem cells [GSCs])^8^ that are characterized by self-renewal, multi-lineage differentiation potential, and the ability to generate xenografts that recapitulate the parental tumors^9^. GSCs have been implicated in tumor infiltration and resistance to radio- and chemotherapy as well as tumor recurrence^10^. GSCs share stemness characteristics with neural stem cells (NSCs) but differ in their differentiation and oncogenic potentials^9,11,12^.

The epithelial-to-mesenchymal transition (EMT) is a process that allows epithelial cells to abandon their polarity and cell-to-cell adhesion properties and acquire mesenchymal cell phenotypes which are associated with enhanced invasiveness, stemness and metastasis, and treatment resistance^13^. Recent studies demonstrated a similar process in glioma, and proneural-to-mesenchymal transition in these tumors is characterized by increased aggressiveness, invasiveness and therapy resistance^14–16^. Mesenchymal transition can occur in recurrent tumors and in response to radiation^17,18^ and is associated with poor patient prognosis.

Tumor aggressiveness and mesenchymal transition of glioma are induced by cells and secreted factors in the tumor microenvironment^19–21^. These cells include endogenous central nervous system (CNS) cells such as astrocytes, oligodendrocytes, neurons and microglia, and infiltrating immune cells^21^. Microglia are resident immune cells in the brain and together with infiltrating macrophages represent the most prevalent CNS-associated cells in the tumors^22^. Microglia and recruited macrophages have been reported to undergo differentiation to cells with M2-type characteristics, in response to factors secreted by glioma cells^23^. The M2 microglia and macrophages further support tumor growth via the secretion of growth factors, chemokines and extracellular matrix-modifying enzymes^22,24^. In addition, recent studies implicated extracellular vesicles (EVs) as important mediators of intercellular communication and in the cross talk of tumor cells and their microenvironments^25^. EVs contain proteins, lipids, and various RNA species and play important roles in the interaction of glioma cells and microglia^23,25^.

The expression of specific microRNAs (miRNAs) has been shown to be associated with several aspects of glioma pathogenesis including cell cycle control, invasion, migration, resistance to therapies, and cell apoptosis^26^. Specific miRNAs have been also implicated in the self-renewal, stemness, and tumorigenic features of GSCs^27^.

Here, we analyzed the miRNA expression of GSCs in comparison with human NSCs (hNSCs) and identified unique miRNA expression profiles that distinguish these two cell populations. Focusing on miR-504, which was highly expressed in hNSCs compared to GSCs, we demonstrated that it was upregulated in the G-CIMP glioma subtype compared to other GBM subtypes. Moreover, we found that miR-504 exerted an antitumor effect in vitro and in vivo and in addition, inhibited the stemness and mesenchymal transit of GSCs. In addition, overexpression of miR-504 in GSCs exerted a bystander effect on cocultured microglia cells by promoting the differentiation of these cells toward M1 phenotype via EV delivery.

## Materials and methods

### GSC cultures

All human materials were used in accordance with the policies of the Henry Ford Hospital Institutional Review Board. Generation of GSCs from fresh GBM specimens and their characterization have been recently described^28–31^. Briefly, the GSCs were maintained in neurosphere medium (DMEM-F12 1:1, glutamine 10 mM, HEPES buffer 10 mM, and sodium bicarbonate 0.025%) supplemented with basic fibroblast growth factor (20 ng/ml) and epidermal growth factor (20 ng/ml). The GSCs expressed markers such as CD44, CD133, Musashi-1, Sox2, and nestin, exhibited self-renewal, and expressed astrocytic and neuronal markers upon differentiation. The GSCs also exhibited tumorigenic potential and generated glioma xenografts in nude mice^28–33^. The full information of the GSCs employed in this study is described in Table S1.

### Microglia and NSC cultures

Human microglial cells were obtained from Applied Biological Material (Richmond, BC, Canada). All cells employed in this study were tested for mycoplasma contamination (Mycoplasma PCR Detection Kit) and found negative. hNSCs (H9, hESC derived) were obtained from Invitrogen.

### Transduction of GSCs and microglial cells

Lentivirus vectors (System Biosciences, Mountain View, CA) expressing the miR-504 reporter, pre-miR-504, miR-504 antagomiR, Grb10, or control and Grb10 shRNAs were packaged and used to transduce the cells according to the manufacturer’s protocol and as previously described^28–30^.

### Neurosphere formation assay

To determine the ability of GSCs to form secondary neurospheres, cells were plated in 24-well plates at a density of 10 and 100 cells/well through limiting dilution and the number of neurospheres/well was determined following 10 days for ten different wells. Spheres that contained more than 20 cells were scored and the results are presented as percentages of maximal neurospheres formed compared to control cells^28,29^.

### In vitro limiting dilution assay

For the in vitro limiting dilution assay, GSCs were plated in 96-well plates in decreasing numbers of cells (50, 20, 10, 5, 2, and 1) per well. Following 10 days, the number of spheres was determined for each well. Extreme limiting dilution was analyzed as recently reported^32^.

### Real-time polymerase chain reaction (RT-PCR)

Total RNA was extracted using RNeasy midi kit according to the manufacturer’s instructions (Qiagen, Frederick, MD). Reverse transcription reaction was carried out using 2-μg total RNA as previously described^28,31^. Briefly, reactions were run on an ABI Prism 7000 Sequence Detection System (Applied Biosystems, Foster City, CA). Cycle threshold (Ct) values were obtained from the ABI 7000 software. S12 levels were used as controls. The primer sequences are described in Table S2.

### Western Blot analysis

Cell pellet preparation and Western Blot analyses were performed as previously described^28–30^.

### Transwell migration assay

Transwell chambers (BD Biosciences, San Jose, CA) were used for analyzing cell migration as recently reported^29,34^ In brief, cells (25,000/well) were incubated for 3 h in culture medium with 10% fetal bovine serum in the bottom chambers. The total number of the migrated cells was determined in fixed and stained cells (0.05% crystal violet for 5 min).

### Cell viability assay

Cells were washed with phosphate-buffered saline (PBS), centrifugated in 3000 rpm for 5 min and the cell pellet was incubated in PBS containing 0.4% trypan to stain the dead cells. The number of Trypan-blue stained cells was determined using a Countess II FL (Thermo Fisher, MA, USA).

### Isolation of GSC-derived EVs

EVs were prepared as previously described^31,35^ using sequential centrifugation at 300 × *g* for 10 min, 2500 × *g* for 20 min, 10,000 × *g* for 30 min and 110,000 × *g* for 90 min. The pellet was then resuspended in PBS and washed twice followed by filtration using a 0.22-μm filter. The protein content of the enriched EV fractions was determined using the Micro BCA assay kit (ThermoFischer Scientific, Oregon City, OR). The expression of the exosome markers CD63, CD81, and CD9 was analyzed by Western blot and the quantification of the isolated EVs was performed using the ExoELISA-Ultra CD63 kit according to the manufacturer’s instructions. For the exosome treatment, 0.5 × 10^8^ EVs were added to the cultured cells.

### ImageStreamX analysis

Microglial cells were treated with GSC-derived EVs labeled with CellTracker Red (ThermoFisher, Waltham, MA) for 24 h. Cells were excited using 561-nm laser, and cell fluorescence of approximately 10^4^ cells per sample was captured and photographed using an ImageStreamX high-resolution imaging flow cytometer (Amnis Co., Seattle, WA) as previously described^35^. The samples were gated to obtain a population of captured single-cell images of living cells, then gated for the cells in focus using the gradient root mean square feature. Cells incubated with or without labeled EVs were compared for the intensity of the red channel fluorescence. Images were analyzed using IDEAS 6.0 software (Amnis Co., Seattle, WA).

### miR-504 reporter

For analyzing miR-504 delivery, a miR-504 luciferase reporter plasmid was employed as previously described for miR-124^36^. A unique miR-504 binding site, which is a fully complementary sequence of mature miR-504, was cloned downstream of luciferase reporter gene of the pMiR-Luc reporter vector from Signosis, Inc. (Santa Clara, CA). For the mCherry reporter, the luciferase gene of pMiR-Luc reporter vector was replaced with mCherry-N1 obtained from Clontech (Mountain View, CA).

### Phagocytosis analysis

Human microglial cells were plated alone or in coculture with GSCs. Phagocytosis was determined using the pHrodo^™^ Green zymosan bioparticle assay (Invitrogen, Carlsbad, CA, USA) according to the manufacturer’s instructions. Briefly, microglia plated alone and in the presence of GSCs were incubated with a solution of pHrodo Green zymosan bioparticles in Live Cell Imaging Solution (0.5 mg/ml). Phagocytosis was determined after 120 min using fluorescence plate reader at Ex/Em 509/533.

### miRNA array processing and analysis

All experiments were performed using Affymetrix HU GENE1.0st oligonucleotide arrays and GeneChip miRNA 4.0 Array (ThermoFisher). Sample processing was performed according to the protocol provided by the company. The rest of the analysis was performed using Partek^®^ Genomics Suite^TM^ software, version 6.6 (^©^2012 Partek, Inc.). miRNA data were summarized using RMA and standardized by sketch-quantile normalization. Differential expression was performed via ANOVA. Significant miRNAs were selected to have at least 1.5-fold change and a *P* value < 0.05. Results were visualized by volcano plot. Functional analysis was conducted by Ingenuity software using the core analysis on differential miRNA lists. The panel of measured miRNAs (a list of all measured miRNAs) was used as the background set for enrichment tests. Networks included up to 35 miRNAs and mRNAs.

### TCGA data analysis

Expression data were downloaded for TCGA cases from the Broad Firehose portal (http://gdac.broadinstitute.org/). GBM cases were assayed by microarray for miRNA expression^6^. The level 3, batch-adjusted, expression data file captured mature miRNA quantification (file date: 12/10/2014). Low-grade glioma (LGG) cases were assayed by miRNA-sequencing^37^. The level 3 data file contained expression data per mature miRNA as reads per million miRNAs mapped (file date: 12/10/2014). GBM and LGG cases were assayed by mRNA-sequencing^5^. The level 3 data file contained RSEM normalized data^38^, quantified per-gene as the normalized count (file date: 12/10/2014). Expression data are used continuously, discretized by quantile, or dichotomized at the median as high/low as appropriate for the research question. Clinical data and molecular classifications were taken from the recent publication of the TCGA Glioma Analysis Working Group^6^. Comparison of mean expression between groups was performed by one-way ANOVA followed by Tukey’s corrected two-sample tests, which adjust for multiple comparisons to maintain the family-wise error rate.

### Xenograft studies

Following the guidelines of Henry Ford Hospital’s Institutional Animal Care and Use Committee, dissociated GSCs (3 × 10^5^ cells) transduced with a lentivirus vector expressing a control pre-miR or pre-miR-504 were inoculated intracranially into nude mice (Nu/Nu) as previously described^32^. Briefly, animals were anesthetized and injected with the GSCs through a 3-mm hole to the right of the bregma at a depth of 2.5 mm and a rate of 5 μL/30 s. All animals were monitored daily and sacrificed at the first signs of neurological deficit.

### Statistical analysis

The results are presented as the mean values ± SD. The data of patient specimens are presented graphically with median and interquartile range noted. Data were analyzed using ANOVA or a Student’s *t* test with correction for data sets with unequal variances. Data were analyzed on a log 2 scale as appropriate. Kaplan–Meier estimates of the survival time from diagnosis until death or last follow-up were used for outcome analysis. Differences in survival curves between groups were assessed by the log-rank test. Cox regression was used to construct multivariable models of survival including miRNA expression, age at diagnosis, IDH mutation status and grade.

## Results

### Functional clustering and networks associated with miRNAs that distinguish GSCs from hNSCs

**Fig. 1:**
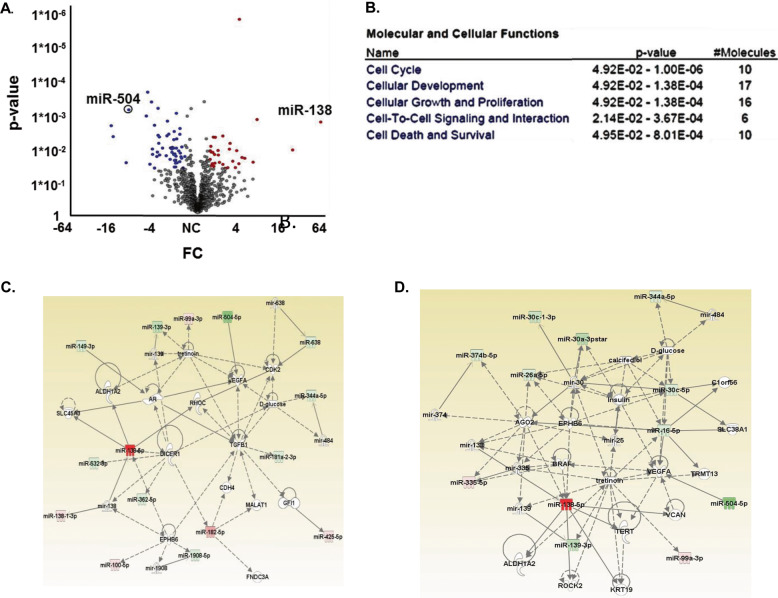
miRNA array analysis of GSCs and hNSCs. miRNA chip array was performed for 12 GSC and 3 neural stem cell NSC cultures. The miRNA profile of GSCs and hNSCs was compared and a volcano plot was generated (**A**). Green circles-miRNAs significantly downregulated in GSCs, red circles-miRNAs significantly upregulated in GSCs. FC > = 1.5, *P* value ≤ 0.05. For these miRNAs, Ingenuity functional enrichment analysis (**B**) and ingenuity networks (**C**, **D**) demonstrated the interconnectedness of the altered miRNAs in GSCs were performed by IPA.

To define the patterns of miRNA expressions that are unique to GSCs and associated with their tumorigenicity and mesenchymal characteristics, we used a miRNA array chip for 12 GSCs and three different cultures of hNSCs. We first compared the miRNA expression of GSCs and hNSCs. miRNAs were identified using cutoffs for ≥1.5-fold differential expression and a significant *P* value (*P* ≤ 0.05), as listed in Table S3 and as shown in a volcano plot (Fig. 1A). Thirty miRNAs were significantly upregulated, and 55 miRNAs were downregulated in GSCs relative to hNSCs. These miRNAs were further analyzed by functional enrichment and network analysis using Ingenuity Pathway Analysis (IPA; Ingenuity Systems, http://www.ingenuity.com). IPA analysis identified clusters of miRNAs that are associated with well-known oncogenic pathways including cell cycle, cellular development, cellular growth and proliferation, cell-to-cell signaling and interaction and cell death and survival (Fig. 1B). IPA was also used to generate three networks of altered miRNA interactions consisting of at least 15 miRNAs from the miRNA lists (Figs. 1C, S1A, B). These networks are associated with miRNA biogenesis including regulation of Dicer1 and AGO2 (Figs. 1C, S1A) and of oncogenes such as TERT, MYC, CASP2, CASP10, BCL2, and TP73 (Fig. S1a). An important oncogenic pathway that was also identified is associated with increased regulation of classical EMT mediators such as Smad2/3, Smad6/7, TGFβ1, and Dicer (Figs. 1C, S1B).

We then identified miRNAs that were expressed in GSCs but not in hNSCs and found that 10 miRNAs were significantly upregulated in GSCs while 37 were downregulated (Fig. S1D). The IPA of these miRNA clusters generated two networks, each containing more than 10 miRNAs (Figs. 1D, S1C). These networks consist of proteins related to cell survival (p53 and TERT) and miRNA biogenesis (Dicer and AGO2) similar to the pathways that were obtained in the initial comparison of the GSCs and hNSCs (Fig. 1D). In addition, the two key mesenchymal markers ZEB2 and RUNX1 were also identified in these networks (Fig. S1C).

### MiR-504 is downregulated in GBMs and GSCs

**Fig. 2:**
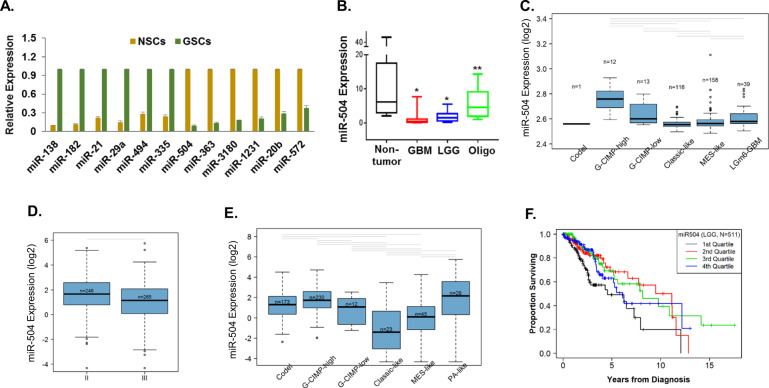
Expression of miR-504 in GSCs and glial tumors. Validation of top miRNAs enriched in GSCs (*N* = 11) compared with the hNSCs (*N* = 4) was performed by RT-PCR (**A**) *P* < 0.001. miR-504 expression in glial tumors compared with normal brains (*n* = 20 for each) was determined using real-time PCR (**B**) *P* < 0.001. Relative expression of miR-504 in GBM (**C**) and LGG (**E**) by subtypes was determined according to The Cancer Genome Atlas (TCGA data portal): gray bars indicate significant differences in post hoc *t* tests *P* < 0.05. Relative expression of miR-504 was also analyzed in grade III and grade II LGG (**D**). Overall survival plotted according to quartile of miR-504 expression among grade II and III glioma (**F**); log-rank *P* = 0.00136 overall; log-rank *P* = 0.00402, 0.00123, 0.0111, Q1 vs. Q2–4, respectively.

Using RT-PCR analysis we first validated some of the miR array results (Figs. 2A, S2). Since miR-504 was one of the most downregulated miRNAs in GSCs compared with hNSCs (Fig. 1A), we focused on this miRNA as a potential inhibitor of the tumorigenicity of GSCs. The expression of miR-504 in GBM specimens was also significantly increased in normal brain compared with astrocytic tumor specimens (Fig. 2B).

We then analyzed the relative expression of miR-504 in the different subtypes of GBM using TCGA^6^. There are 339 GBM cases in our study that have IDH/Methylation subtyping. This subtyping splits the IDHmut-noncodel class into two groups according to methylation pattern (Fig. 2C). The G-CMIP-low class has a lower level of methylation globally and has been found to have worse prognosis. The IDHwt GBM tumors are split into three groups. Two align with expression class, as mesenchymal-like and classic-like, and the third has a distinct methylation pattern, denoted as LGm6–GBM (Fig. 2C, ANOVA, *P* < 0.0001). Gray lines indicate significant difference between groups (post hoc *t* test, *P* < 0.05). Analysis of miR-504 expression in LLGs demonstrated that the expression of miR-504 was higher on average in grade II glioma compared with grade III (Fig. 2D).

There are 509 LGG cases in our study that have IDH/methylation subtyping. This subtyping splits the IDHmut class into three groups according to methylation pattern (Fig. 2E). The G-CMIP-high class has a highest miR-504 expression. The IDHwt LGG tumors are also split into three groups. The classic-like and PA-like are two groups with the lowest and highest expression, respectively. While the mesenchymal-like group has intermediate expression (Fig. 2E). Gray lines indicate significant difference between groups (post hoc *t* test, *P* < 0.05). While survival differences were observed by IDH-mutation status (data not shown), there was no evidence that miR-504 expression has independent prognostic value beyond the two new WHO 2016 diagnostic groups, GBM with IDHwt and GBM with IDH mutant. A Kaplan–Meier plot demonstrates that the quarter of patients with lowest expression of miR-504 has the worst survival outcome among grade II and III glioma (Fig. 2F; log-rank *P* = 0.00136 overall; log-rank *P* = 0.00402, 0.00123, 0.0111 Q1 vs. Q2–4, respectively). Collectively, these results indicate that miR-504 expression is inversely correlated with tumor aggressiveness and poor prognosis.

### miR-504 inhibits the stemness and mesenchymal transit of GSCs

**Fig. 3:**
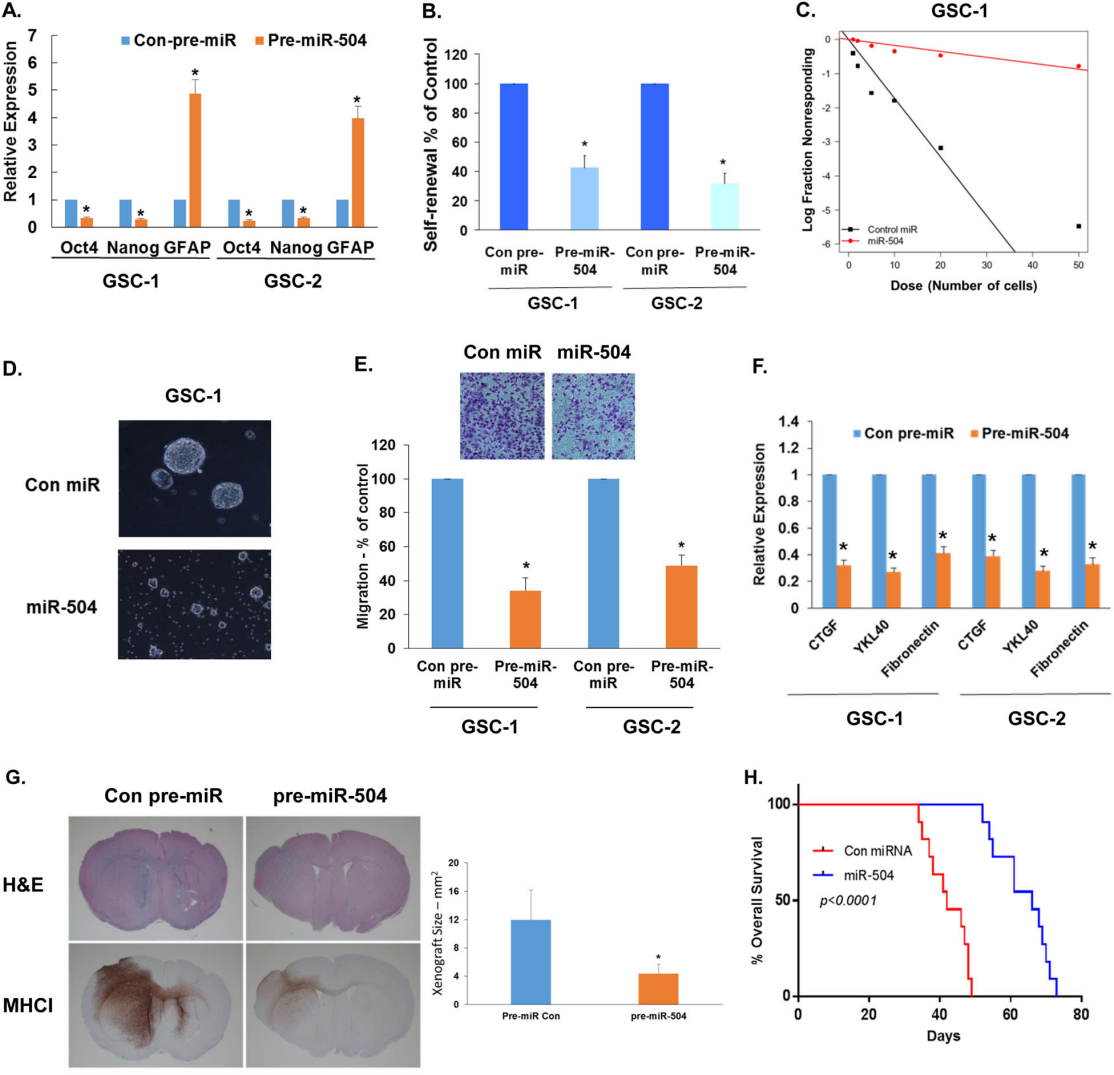
miR-504 inhibits stemness, mesenchymal markers and oncogenic potential in GSCs. GSC-1 and GSC-2 were transduced with lentivirus vectors expressing control pre-miR or pre-miR-504 and the expression of stemness markers was determined using RT-PCR *P* < 0.001 (**A**). Self-renewal analysis was performed with the two different GSCs. Cells overexpressing a control or miR-504 pre-miRs were plated at 10 cells/well in 96-well plates and the number of neurospheres per well was quantified after 10 days. *P* < 0.0001 (**B**). In vitro extreme limiting dilution assay (ELDA) demonstrated that overexpression of pre-miR-504 decreased the frequency of neurosphere formation in GSC-1 (**C**). Representative pictures of neurospheres (GSC-1) after 2 weeks in culture are presented (**D**). The results represent at least three different experiments/samples that gave similar results. GSCs overexpressing pre-miR-504 were also analyzed for cell migration using the transwell migration assay (**E**) and for the expression of mesenchymal markers using real time PCR *P* < 0.001 (**F**). GSC-1 overexpressing a control pre-miR or miR-504 were implanted intracranially into nude mice and tumor size was determined in brain sections stained for H&E and for human anti-MHCI antibody following 4 weeks of transplantation (**G**) *P < 0.001. Kaplan–Meier survival curves for mice bearing intracranial xenografts derived from GSC-1 overexpressing pre-miR-504 or control pre-miR (*n* = 11) were determined by both log-rank (Mantel–Cox) test and Gehan–Breslow–Wilcoxon test (**H**) *P* < 0.0001.

To examine the effect of miR-504 we overexpressed it in GSCs (Fig. 3SA) and found that pre-miR-504 markedly decreased the expression of the stemness markers Oct4 and Nanog and increased the expression of the astrocytic marker GFAP in both GSC-1 and GSC-2 (Fig. 3A).

The role of miR-504 in the stemness of GSCs was further examined on the self-renewal ability and neurosphere formation of these cells. Overexpression of miR-504 in GSCs decreased their ability to form neurospheres as indicated by analyzing secondary neurosphere formation (Fig. 3B), the extreme limiting dilution assay (Fig. 3C) and spheroid size (Fig. 3D), suggesting that miR-504 inhibited the stemness potential of GSCs and increased their differentiation. miR-504 overexpression did not induce cell death in the GSCs as determined by trypan blue staining (data not shown).

In addition, overexpression of miR-504 also decreased GSC migration (Fig. 3E) and the expression of the mesenchymal markers CTGF, fibronectin 1 (FN), and YKL-40 (Fig. 3F). These results demonstrate that miR-504 acts as a negative regulator of the stemness, mesenchymal phenotypes, and migration of GSCs.

We further examined the effects of miR-504 overexpression on the tumorigenic capacity of GSCs in vivo. For these experiments we employed GSC-1 transduced with lentivirus vectors expressing pre-miR-504. Intracranial implantation of these cells into immunocompromised mice resulted in significantly smaller xenografts (4.35 ± 1.33 mm^2^; *n* = 5) compared with those expressing a control pre-miR (11.96 ± 4.20 mm^2^; *N* = 5, *P* < 0.005) (Fig. 3G). In addition, overexpression of miR-504 increased the mean survival of tumor-bearing mice compared with the control miR group (66 days vs. 42 days, *P* < 0.0001, *N* = 11/group) (Fig. 3H). These data demonstrate that overexpression of miR-504 in GSCs decreased their tumorigenicity which is in line with the favorable prognosis of patients with tumors that express high levels of this miR.

### Grb10 is a target of miR-504 and mediates the inhibitory effects of this miR on GSCs

**Fig. 4:**
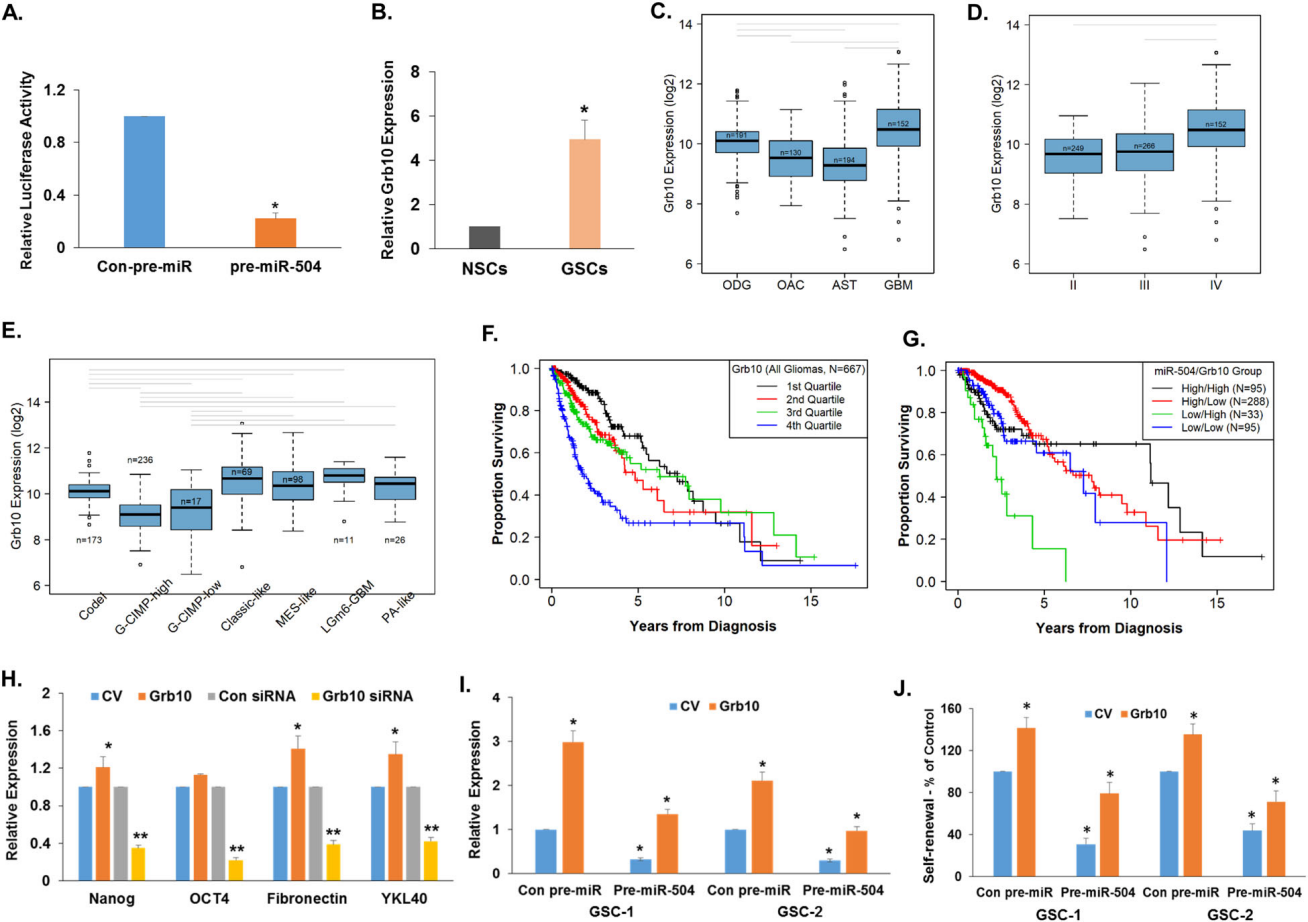
Targeting of Grb10 mediates the anti-tumor effects of miR-504. The direct targeting of Grb10 by miR-504 was determined using luciferase assay of the Grb10 3′-UTR luciferase plasmid *P* < 0.001 (**A**). The expression of Grb10 was determined in hNSCs (*N* = 4) and GSCs (*N* = 11) by RT-PCR *P* < 0.01 (**B**). The expression of Grb10 in the different subtypes of glioma was determined using data from TCGA. Boxplots of Grb10 expression are shown on a log-2 scale by histology (WHO2007 criteria); (ODG oligodendroglioma, OAC oligoastrocytoma, AST astrocytoma, GBM glioblastoma) (**C**), by WHO Grade (**D**), and by methylation subclass (**E**). Gray lines indicate a significant difference between groups (post hoc *t* test, *P* < 0.05). A Kaplan–Meier plot (**F**) shows that the quarter of patients with highest expression of Grb10 have the worst survival outcomes (log-rank *P* = 6.39e−12 overall; log-rank *P* = 5.13e−11, 6.15e−07, 2e−05, Q4 vs. Q1–3). Overall survival plotted according to high/low expression of miR-504 and Grb10 dichotomized at 25th percentile for miR-504 and 75th percentile for Grb10 to provide a better separation. log-rank *P* = 1e−04 (**G**). Median survival is indicated on the graphs. GSC-1 cells were transduced with lentivirus vectors expressing a control vector, Grb10, a control shRNA or Grb10 shRNA, and the expression of mesenchymal and stemness markers was analyzed using RT-PCR (**H**) *P < 0.005; **P < 0.01. The role of Grb10 in miR-504 effects on Nanog expression (**I**) and self-renewal (**J**) was analyzed in GSCs transduced with lentivirus expressing miR-504 with and without Grb10 lacking the 3′-UTR. *P < 0.001. The results represent at least three different experiments that gave similar results.

Bioinformatics analysis identified Grb10 as a potential target of miR-504 which was also recently reported as a validated one^39^. Using the Grb10 3′-UTR- tagged to luciferase, we demonstrated a direct targeting of Grb10 by miR-504 (Fig. 4A). We then analyzed the expression of Grb10 in hNSCs and GSCs (*N* = 10) and found that this gene was highly expressed in GSCs compared to hNSCs (Fig. 4B). Using TCGA we analyzed the expression of Grb10 in various subtypes of glioma and found that the expression of Grb10 was significantly higher in GBM compared with low-grade tumors as determined by histology criteria (Fig. 4C) and the WHO grade (Fig. 4D). In addition, analysis of methylation glioma subtypes demonstrated that Grb10 was highly expressed in the mesenchymal subtype and exhibited lowest expression in the G-CIMP high tumors (Fig. 4E). Kaplan–Meier analysis (Fig. 4F) shows that the quarter of patients with highest expression of Grb10 have the worst survival outcomes (log-rank *P* = 6.39e−12 overall; log-rank *P* = 5.13e−11, 6.15e−07, 2e−05, Q4 vs. Q1–3, respectively).

Dichotomizing miR-504 at the 25th and Grb10 at the 75th percentiles, demonstrates that the low/high pattern of miR-504 and Grb10, respectively, more clearly identifies a subset of patients with poor overall survival compared to each expression alone (Fig. 4G, log-rank *P* = 2.67e−8 overall; log-rank *P* = 0.0012, 2.89e−12, 0.0003, low/high vs. high/high, high/low and low/low, respectively).

We then examined the role of Grb10 in GSC functions and demonstrated that its overexpression in GSCs increased (Fig. 4H), while its silencing decreased (Fig. 4H) the stemness and mesenchymal phenotypes of GSCs, similar to the effects of miR-504. Overexpression of a Grb10 plasmid lacking 3′-UTR induced a modest upregulation of self-renewal of the GSCs and partially abrogated the inhibitory effect of miR-504 on the mesenchymal phenotype (Fig. 4I) and the self-renewal (Fig. 4J) of the treated GSCs. These results demonstrate that targeting Grb10 by miR-504 mediates at least in part the inhibitory effects of miR-504 on the stemness and mesenchymal phenotypes of GSCs.

### Overexpression of miR-504 in GSCs promotes M1 (pro-inflammatory) phenotypes of cocultured microglial cells

Glioma cells and GSCs have been demonstrated to induce polarization of microglia toward the M2 phenotype (anti-inflammatory/pro-tumorigenic) by the secretion of cytokines and EV-derived miRNAs^23,40,41^. In addition, we recently reported that EVs can deliver exogenous miRNAs both in vitro and in vivo^31,36^. We therefore hypothesized that the overexpressed miR-504 in GSCs can be transferred to neighboring cells and therefore affects not only the oncogenic functions of the GSCs but also their interactions with neighboring cells such as microglia.

**Fig. 5:**
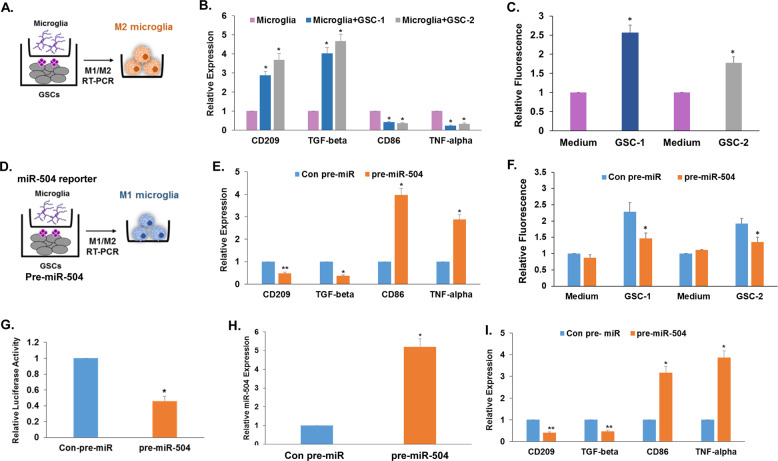
Overexpression of miR-504 in GSCs induces M1 polarization of cocultured human microglia. GSC-1 cells and microglial cells were cocultured in transwell plates with 1-μM filters (**A**). The expression of M1 and M2 markers in microglia was determined by RT-PCR (**B**) and phagocytosis by the pHrodo^™^ Green zymosan bioparticle assay (**C**). GSC-1 cells transduced with lentivirus vectors expressing a control pre-miR or pre-miR-504 were cultured in transwell plates with microglial cells transduced with lentivirus vectors expressing a miR-504 reporter tagged to luciferase (**D**). The expression of the M1 and M2 markers in microglia was determined using RT-PCR (**E**) and phagocytosis by the pHrodo^™^ assay (**F**). The luciferase activity of the miR-504 reporter was also determined (**G**). The expression of miR-504 in the cocultured microglial cells was analyzed using RT-PCR (**H**). Overexpression of miR-504 microglia increased the relative expression of the M1 markers and decreased those of M2 as determined by RT-PCR (**I**). The results represent at least three different experiments/samples that gave similar results. *P < 0.001, **P < 0.01.

For these studies we employed co-cultures of microglial cells and GSCs overexpressing pre-miR-504 or a control pre-miR in transwell plates with 1-μm filters that allow only the transfer of soluble factors and EVs as described in Fig. 5A. Coculturing of microglia with control GSCs or cells transduced with lentivirus expressing a control pre-miR (Fig. 5A, D) resulted in a relative increased expression of the M2-associated markers, CD209 and TGF-β and in the decreased expression of CD86 and TNF-α (Fig. 5B, E). GSC coculturing also increased the phagocytosis of microglia cells (Fig. 5C). In contrast, transduction of the GSCs with pre-miR-504 (Fig. 5D) decreased the expression of the M2-associated markers, increased the expression of CD86 and TNF-α (Fig. 5E) and decreased phagocytosis (Fig. 5F). To verify that miR-504 was delivered by the GSCs to the cocultured microglial cells, we employed a miR-504 reporter tagged to luciferase that allows the quantification of the transferred miRNA as was previously reported^33^. Microglial cells were transduced with a lentivirus vector expressing the miR-504-luciferase reporter and the GSCs were transduced with lentivirus vector expressing either control pre-miR or pre-miR-504 (Fig. 5D). As presented in Fig. 5G, microglia that were co-cultured with GSCs overexpressing pre-miR-504 exhibited decreased luciferase activity indicating that miR-504 was transferred by the cocultured GSCs. These results indicate that miR-504 was transferred by GSCs to cocultured microglial cells. We further analyzed the expression of miR-504 in the cocultured microglial cells and found that they expressed high levels of this miR compared with microglia cocultured with GSCs expressing a control miR (Fig. 5H). Finally, we demonstrated that overexpression of miR-504 in microglial cells upregulated the relative expression of the M1 markers CD86 and TNF-α (Fig. 5I).

### EV-associated miR-504 derived from GSCs induces microglia M1 phenotypes

**Fig. 6:**
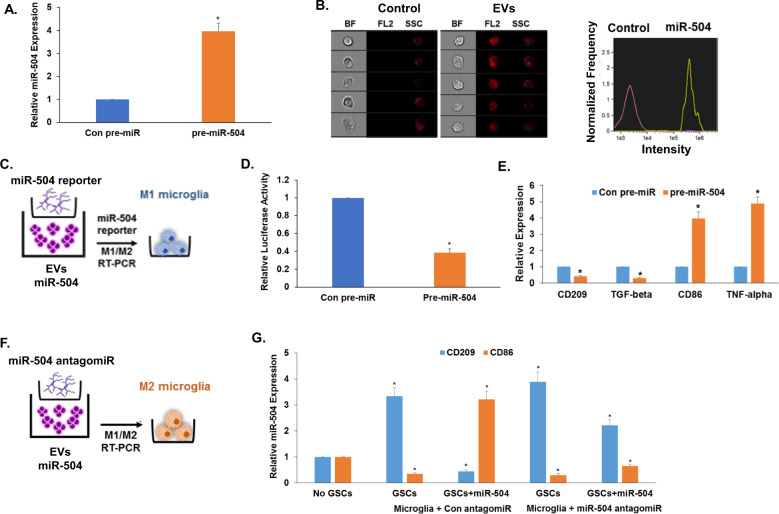
Exogenous miR-504 is delivered by GSC-derived EVs to cocultured microglial cells and promotes their M1 polarization. The expression of miR-504 in EVs derived from GSC-1 cells was analyzed by RT-PCR (**A**). Fluorescence intensity analysis of microglial cells incubated with CellTracker Red labeled EVs isolated from GSC-1 cells compared with untreated cells. A representative image is presented (**B**). EVs derived from GSC-1 overexpressing a control pre-miR or pre-miR-504 were added to human microglia expressing the miR-504 reporter (**C**). The luciferase activity of the miR-504 reporter was measured (**D**) and the expression of M1 and M2 markers in the microglia was determined by RT-PCR (**E**). Microglia cells expressing control or miR-504 antagomiRs were incubated with EVs derived from GSC-1 cells overexpressing miR-504 (**F**) and the expression of the M1 and M2 markers were analyzed by RT-PCR (**G**). The results are representative of at least three different experiments that gave similar results. For statistical analysis, *P < 0.001.

EV-associated miRNAs are implicated in the cross-talk of GSCs and microglia^33,35^. To determine the role of EVs in the delivery of miR-504 to microglial cells, we first analyzed the expression of miR-504 in EVs derived from GSC-1 that were transduced with lentivirus vectors expressing control pre-miR or pre-miR-504. EVs were isolated from GSC-1 cultures using differential ultracentrifugation as previously described^35^ and were analyzed for the expression of CD63, CD81, and CD9 (Fig. S3B). The amount of the secreted EVs was determined using ELISA of CD63 antibodies and was found to be comparable in GSC-1 overexpressing con-miR or miR-504 (data not shown). We found that EVs isolated from GSC-1 overexpressing miR-504 expressed significantly higher levels of miR-504 compared with EVs isolated from GSC-1 expressing a control pre-miR (Figs. 6A, S3C). We next demonstrated the transfer of EVs from GSC-1 to microglial cells using ImageStreamX analysis. In these studies, GSC-1 derived EVs labeled with CellTracker Red were added to microglial cells and their fluorescence was determined 12 h later. The EVs were efficiently internalized and accumulated in the microglial cells (Fig. 6B). Incubation of microglial cells that express the miR-504 reporter with EVs that were isolated from GSC-1 expressing either the con-miR or miR-504 (Fig. 6C) demonstrated the functional delivery of the miR-504 to the microglial cells (Fig. 6D), which resulted in decreased expression of CD209 and TGF-β, and increased expression of CD86 and TNF-α (Fig. 6E), similar to our observations with cocultured GSCs (Fig. 5D). To demonstrate that the delivered miR-504 mediated the effects of the GSC-1 derived EVs, we examined the effects of EVs isolated from GSC-1 overexpressing miR-504 on the differentiation of microglial cells transfected with a miR-504 antagomir (Fig. 6F) and found that these effects were markedly abrogated, whereas, no significant inhibition was observed in microglia transfected with a control antagomir (Fig. 6G). These results indicate that the transfer of miR-504 by GSC-derived EVs mediated, at least partly, the increased M1 phenotypes of the microglial cells induced by the cocultured GSCs.

## Discussion

GBM is one of the most aggressive, infiltrative and incurable tumors with an average patient survival of around 14–16 months^1,2^. GBM therapy resistance and recurrence are primarily attributed to the existence of GSCs^8–10^. Therefore, targeting these cells is an essential component of any successful therapeutic approach. Recent studies demonstrated that the mesenchymal differentiation of GBM is associated with acquisition of stemness characteristics, tumor aggressiveness, therapy resistance, and poor clinical outcome^16–18,42^. Thus, deciphering the mechanisms underlying the mesenchymal differentiation of GSCs is essential for identifying novel therapeutic targets and improving patient survival.

miRNAs have emerged as attractive therapeutic targets due to their critical roles in the regulation of major cell processes such as cell proliferation, stemness, and apoptosis that are key components in cancer initiation and progression^29–31,43^. In addition, specific miRNAs have been implicated in controlling the mesenchymal differentiation of tumor cells^26,44,45^.

Using miRNA microarray analysis of GSCs in comparison to hNSCs, we identified 85 miRNAs that were significantly altered in the GSCs compared with hNSCs. These miRNAs were found to be associated with well-known tumorigenic pathways including cell cycle, cellular development, cellular growth and proliferation, cell-to-cell signaling and interaction, and cell death and survival. These findings indicate that alterations in miRNA expression are associated with deregulation in pathways which contribute to the tumorigenic phenotypes of GSCs.

Additional differences in miRNA expression between GSCs and hNSCs were also associated with the EMT process and included pathways regulating Smad2/3, Smad6/7 TGFβ1^46^, and Dicer^47^, suggesting that the expression of specific miRNAs in GSCs regulate their own expression in parallel with the tumorigenic characteristics of these cells. Finally, other IPA networks identified ZEB2 and RUNX1, two major regulators of the EMT pathway^7^, as mainly enriched in GSCs.

One of the most downregulated miRNAs in GSCs compared to hNSCs was miR-504. We further found that miR-504 expression was significantly lower in GBM as compared to normal brains and exhibited a grade-dependent expression. In addition, using the TCGA portal, we found that miR-504 expression was significantly increased in the G-CIMP high glioma and more generally in IDH-mutant GBM, whereas, it was considerably lower in the IDH-wt glioma classes including the mesenchymal-like subtypes. In agreement with the lower expression of miR-504 in more high grade tumors and in the mesenchymal subtype, we found that overexpression of miR-504 inhibited the self-renewal and mesenchymal phenotypes of GSCs, Collectively, the current results highlight miR-504 as a potential tumor suppressor miRNA and as a negative regulator of the tumorigenicity of GBM and GSCs.

Our results of a role of miR-504 as a tumor suppressor in glioma and as an inhibitor of mesenchymal transformation are in agreement with recent publications^48–52^. The current studies present new data regarding the expression of miR-504 in patient-derived GSCs compared with NSCs and in specific subtypes of gliomas including patient survival data. In addition, the current studies focus on the functions of miR-504 in GSCs including their tumorgenicity using intracranial xenografts.

The role of miR-504 in oncogenic processes appears to be tumor dependent. Thus, in gastric cancer miR-504 expression was decreased by the tumor suppressor gene Trefoil factor 1 (TFF1) that leads to the activation of p53^53^, whereas miR-504 had a dual function in oral squamous cell carcinoma^54^. Moreover, serum expression of miR-504 were demonstrated to differentiate between primary and metastatic brain tumors^55^, suggesting a role of this miR as a diagnostic marker and a mediator of the interaction of glioma and non-CNS cells.

The inhibitory effects of miR-504 on GSCs were at least partly mediated by Grb10, which was recently reported as a validated miR-504 target in vascular endothelial cells^39^. Grb10 is an imprinted gene that is differentially expressed from two promoters and in the brain it is paternally expressed^56^. The role of Grb10 in tumorigenesis is just beginning to be understood^57,58^. Using TCGA analysis, we demonstrated that Grb10 was highly expressed in more aggressive glioma tumors and its expression was directly correlated with worse prognosis. In addition, overexpression of Grb10 in GSCs promoted their aggressiveness, whereas silencing exerted an opposite effect and abrogated the inhibitory effect of miR-504. Thus, our studies demonstrated the miR-504/Grb10 pathway as an important regulator of the stemness-EMT process in GSCs.

Additional targets of miR-504 were reported in glioma and a recent study reported that miR-504 inhibited EMT by targeting the Frizzled-7-mediated the Wnt-β-catenin pathway^51^.

GSCs have been reported to promote the differentiation of microglia toward the M2/anti-inflammatory phenotype, an effect that is more pronounced in mesenchymal GSCs^23,40,41^. The M2 microglia phenotype in turn acts to support the migration and aggressiveness of the tumor cells and inhibition of anti-tumor immune response^24,59,60^. The cross talk between microglia and GSCs is mediated by secreted cytokines and EV-derived miRNAs^23,40,41,61^ In view of our recent reports that EVs can also deliver exogenous miRNA to neighboring cells^31,36^, we hypothesized that overexpression of miR-504 in GSCs may be transferred to neighboring cells via EVs. Using a miR-504 reporter that can directly detect changes in miRNA levels, we found that GSCs overexpressing miR-504 delivered this miRNA to cocultured microglia via EVs. We also found that the increased relative expression of M2 markers induced by GSCs was abrogated in microglia co-cultured with GSCs overexpressing a miR-504 mimic.

The mechanisms by which the EV-derived miR-504 exerts its effects on the microglial cells are currently being studied. Since miR-504 decreased the stemness and mesenchymal differentiation of GSCs, it is possible that other factors or miRNAs that are secreted by the transduced GSCs can also contribute to the induction of this change in the microglial phenotype.

**Fig. 7:**
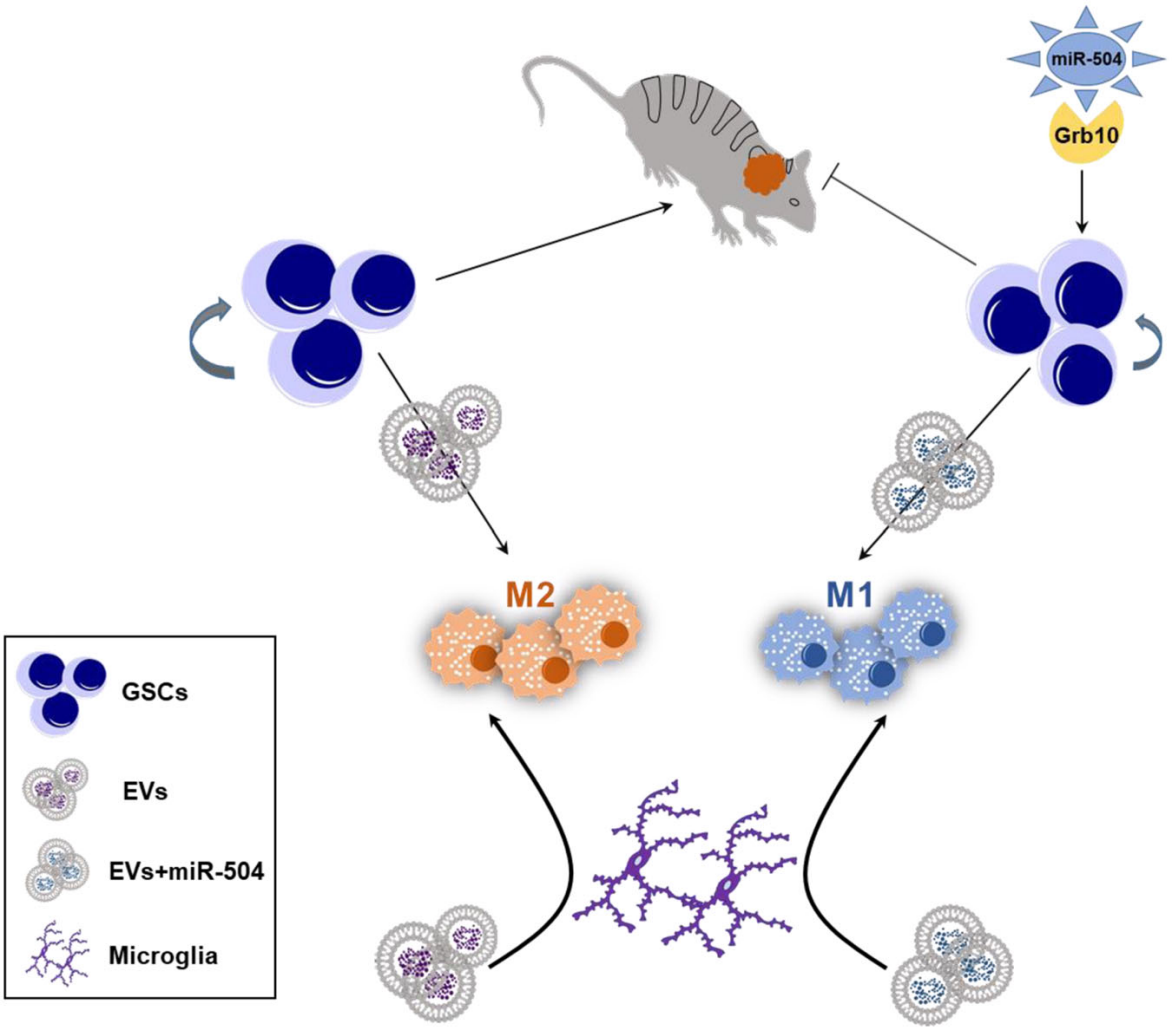
A diagram summarizing the role miR-504 on GSCs and microglia polarization via delivery by EVs. The effects of miR-504 on GSCs and microglia polarization are depicted in this diagram.

The mutual crosstalk between glioma cells and microglia highlights the importance of soluble factors as potential therapeutic targets. Indeed, EV-associated miRNAs have been reported to play an important role in intercellular interactions in both physiological and pathological conditions^62,63^. In addition, overexpressed miRNA mimics have been also reported to be delivered by EVs to neighboring cells^36,39^. Our data indicate that overexpressing miR-504 in GSCs affects not only the functions of the tumor cells but also the tumor-promoting activity of microglia and probably macrophages in the tumor microenvironment, thereby amplifying the therapeutic effect of miR-504 (Fig. 7).

## Conclusions

The acquisition of mesenchymal phenotypes has been associated with increased stemness, infiltration, and aggressive phenotypes in GSCs^64^. Therefore, identifying therapeutic targets that can interfere with this process is of utmost importance. Performing comparative analyses of hNSCs and GSCs, we identified novel miRNAs and potential target networks that are associated with the stemness and mesenchymal transit of GSCs. miR-504 is downregulated in GSCs and exerts inhibitory effects on the functions of these cells via the targeting of Grb10 that acts as an oncogene in GBM and GSCs. Importantly, the overexpression of miR-504 in GSCs not only inhibits the tumorigenic potential of GSCs in vitro and in vivo but can be also transferred to microglial calls and promote their M1 polarization. Thus, the antitumor effects of RNA-based therapy in cancer cells can further exert a bystander effect on the tumor microenvironment via EV delivery.

## Supplementary information

Supplementary Figure S1

Supplementary Figure S2

Supplementary Figure S3

Supplementary figure legends

Supplementary tables

## Data Availability

Data are available in the Supplementary files.
