# Supplementary figures and images for: miR-504 modulates the stemness and mesenchymal transition of glioma stem cells and their interaction with microglia via delivery by extracellular vesicles
Source: Cell Death Dis. 2020 Oct 22;11(10):899. doi: 10.1038/s41419-020-03088-3 (PMC7581800; doi:10.1038/s41419-020-03088-3)

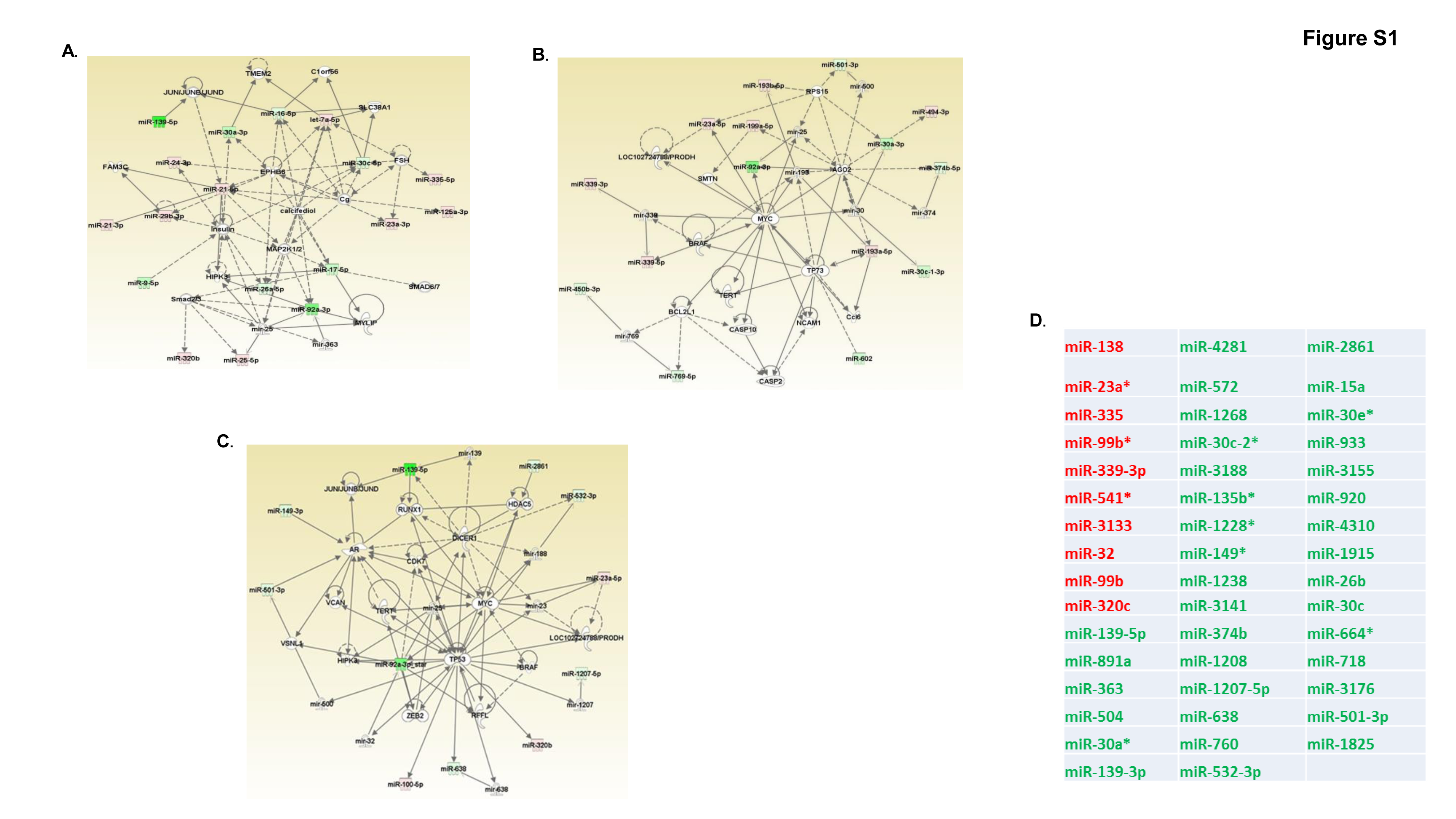

Supplement: Supplementary file 1 — Supplementary Figure S1 [file 41419_2020_3088_MOESM1_ESM.tif]

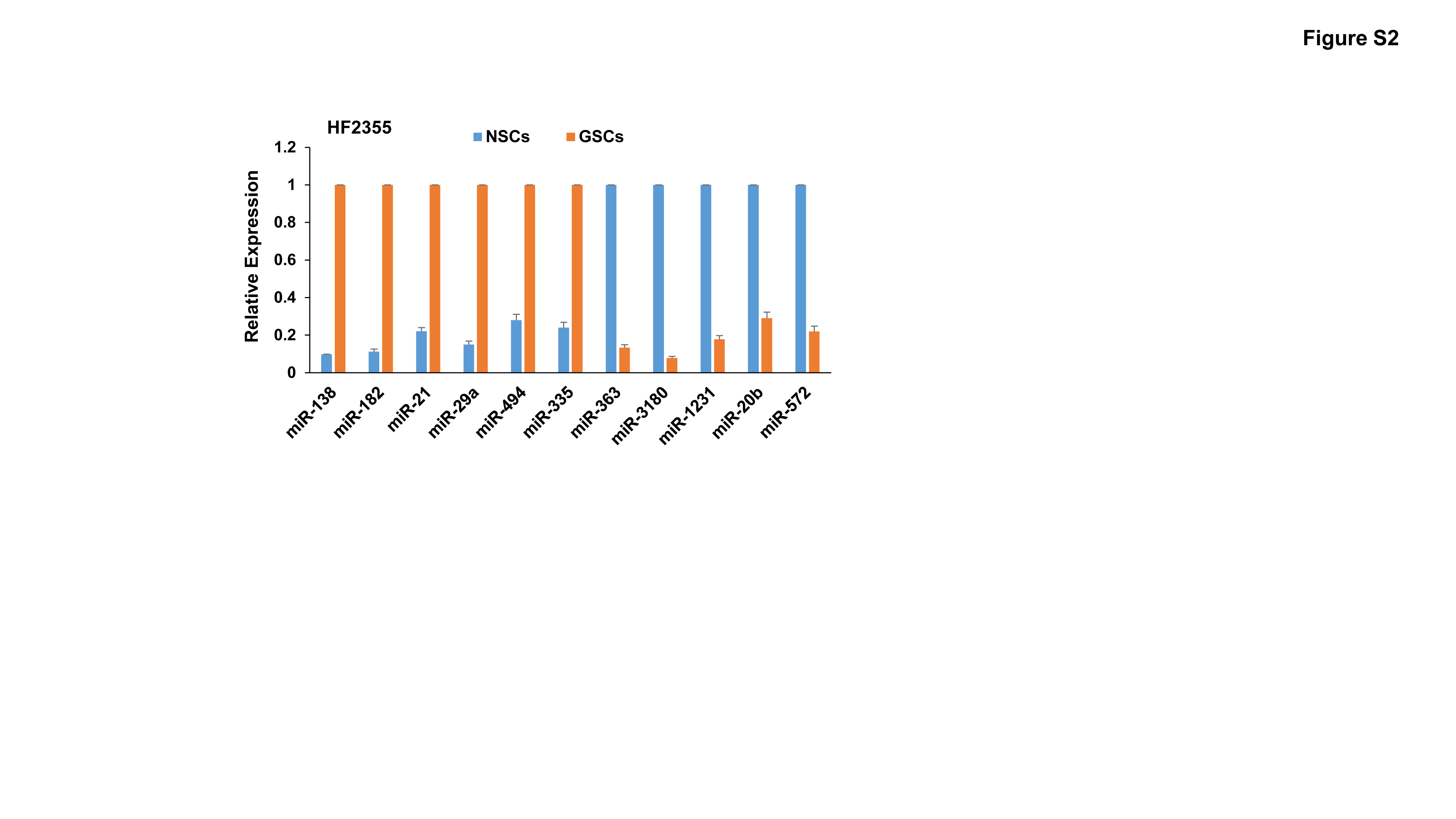

Supplement: Supplementary file 2 — Supplementary Figure S2 [file 41419_2020_3088_MOESM2_ESM.tif]

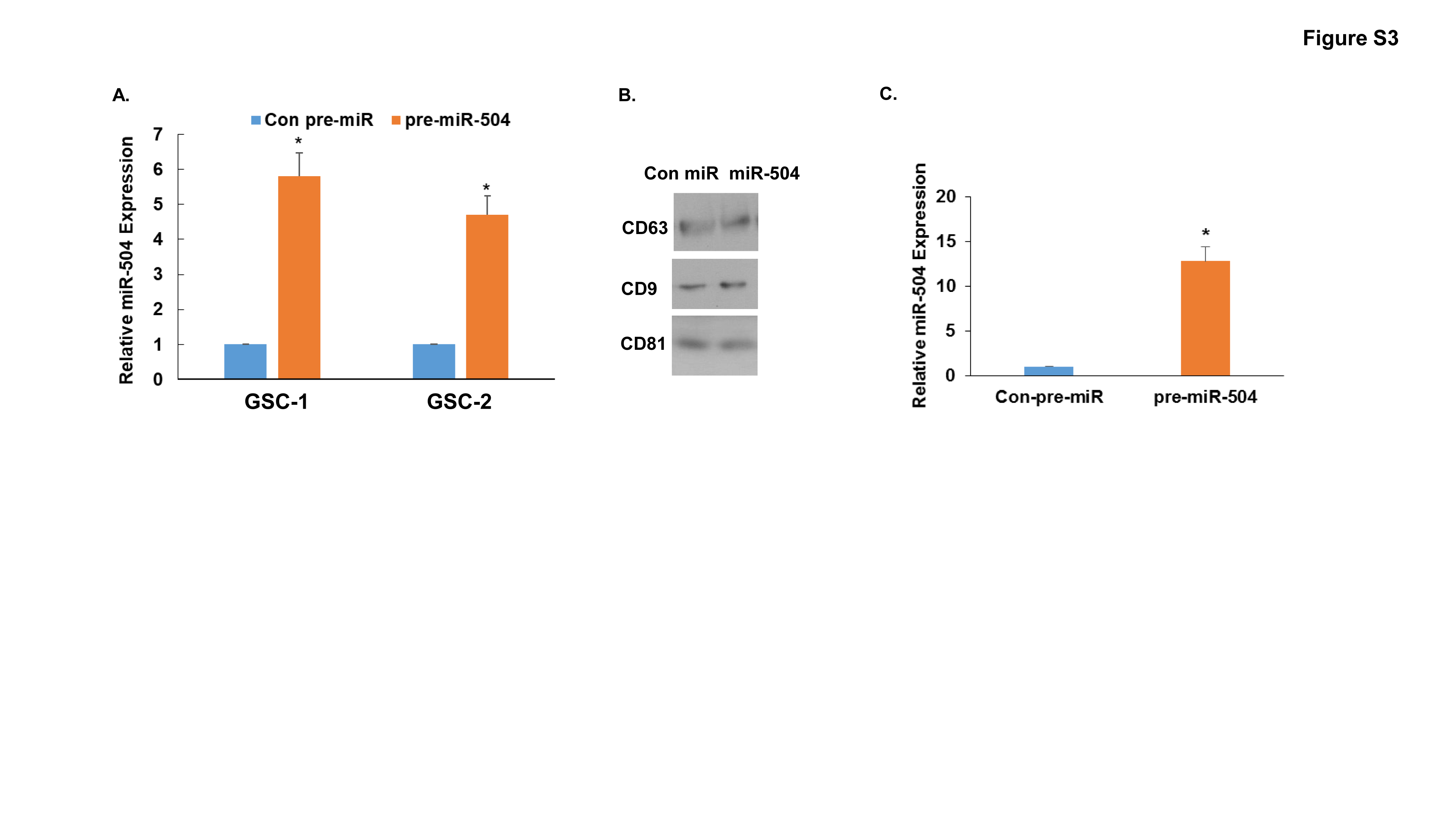

Supplement: Supplementary file 3 — Supplementary Figure S3 [file 41419_2020_3088_MOESM3_ESM.tif]
