# Supplementary material for: miR-504 modulates the stemness and mesenchymal transition of glioma stem cells and their interaction with microglia via delivery by extracellular vesicles
Source: Cell Death Dis. 2020 Oct 22;11(10):899. doi: 10.1038/s41419-020-03088-3 (PMC7581800; doi:10.1038/s41419-020-03088-3)
Supplement: Supplementary file 4 — Supplementary figure legends [file 41419_2020_3088_MOESM4_ESM.docx]

**Supplementary figure and table legends**

**Figure S1. miRNA array analysis of GSCs and NSCs**. Ingenuity network analysis demonstrates novel pathways of altered miRNAs in GSCs compared to NSCs (A-C). List of miRNAs differentially expressed in GSCs and NSCs (D).

**Figure S2. Validation of top miRNA expression in GSCs and NSCs.** Validation of top miRNAs enriched in the GSC-2 compared with the NSCs was performed by RT-PCR. P<0.001

**Figure S3. Overexpression of miR-504 in GSCs and their secreted EVs.** GSC-1 and GSC-2 were transduced with lentivirus vectors expressing a control pre-miR or pre-miR-504 and the expression of miR-504 was determined after 4 days using qRT-PCR (A). EVs were isolated from the GSC-1 using ultracentrifugation and the expression of specific markers was analyzed using Western blot analysis (B). The expression of miR-504 in the GSC-derived EVs was determined using qRT-PCR (C). P<0.001.

**Table S1. De-identified patient information of GSCs.**

For each GSC (n =11), the age, gender, survival data, MGMT (U-unmethylated and M-methylated) and p53 status are presented. Mesenchymal phenotypes of the GSCs were determined by the relative expression of YKL40, SMA and GTGF.

**Table S2. Sequences of primers used for RT-PCR**

**Table S3. miRNAs differentially expressed in GSCs compared to NSCs.** miRNA lists significantly altered in GSCs compared to NSCs. Fold change>=1.5, p<=0.05.
