# Supplementary material for: miR-504 modulates the stemness and mesenchymal transition of glioma stem cells and their interaction with microglia via delivery by extracellular vesicles
Source: Cell Death Dis. 2020 Oct 22;11(10):899. doi: 10.1038/s41419-020-03088-3 (PMC7581800; doi:10.1038/s41419-020-03088-3)
Supplement: Supplementary file 5 — Supplementary tables [file 41419_2020_3088_MOESM5_ESM.docx]

**Table S1. De-identified patient information of GSCs**

| **Cell Line** | **Age@diagnosis**  **(years)** | **Gender** | **OS (days)** | **MGMT** | **P53 mutation status** | **Mesenchymal markers** |
| --- | --- | --- | --- | --- | --- | --- |
| **GSC-1** | 66 | M | 189 | M | wt | H |
| **GSC-2** | 54 | M | 432 | M | wt | H |
| **GSC-3** | 66 | F | 1481 | U | wt | L |
| **GSC-4** | 45 | F | 45 | U | wt | H |
| **GSC-5** | 62 | M | 558 | U | wt | H |
| **GSC-6** | 54 | F | 339 | U | R175H | H |
| **GSC-7** | 67 | F | 131 | U | wt | H |
| **GSC-8** | 79 | F | 138 | U | C242F | H |
| **GSC-9** | 59 | M | 270 | M | V272M | H |
| **GSC-10** | 39 | F | 717 | M | wt | L |
| **GSC-11** | 45 | M | 646 | U | M133T | L |

For each GSC (n =11), the age, gender, survival data, MGMT (U-unmethylated and M-methylated) and p53 status are presented. Mesenchymal phenotypes of the GSCs were determined by the relative expression of YKL40, SMA and GTGF.

**Table S2. Sequences of primers used for RT-PCR**

| **Gene** | **Sequence** |
| --- | --- |
| **IL-13** | S: CGAGAAGACCCAGAGGATG  AS: GTCTCGGACATGCAAGCTG |
| **TNF-α** | S: CCACGCTCTTCTGCCTGCT  AS: GCTTGTCACTCGGGGTTCG |
| **IL-1α** | S: CCAAGATGAAGACCAACCA  AS: GCCAAGCACACCCAGTAGT |
| **GFAP** | S: TCCTTGACCTGCGACCTG  AS: TCTGCCCCTCTTCCTCCA |
| **CD68** | S: ACAATGTGTCCTTCCCCCAC  AS: CCGATGATGAGAGGCAGCAA |
| **CD206** | S: CATCAGGGTGCAAGGAAGG  AS: GTCCAGGCACTGAAAGTGGA |
| **CTGF** | S: GTGTGCACCGCCAAAGATG  AS: CAACCACGGTTTGGTCCTTG |
| **YKL40** | S: TGCCCTTGACCGCTTCCTCT  AS: TTGATGAAAGTCCGGCGACT |
| **Fibronectin** | S: CGAGGAGAGTGGAAGTGTGAGAG  AS: GTGAGGCTGCGGTTGGTAAA |
| **OCT4** | S: ATCAGCCACATCGCCCAGCA  AS: CCCAGCAGCCTCAAAATCCT |
| **Nanog** | **S:** ACCTATGCCTGTGATTTGTGG  AS: GTTGTTTGCCTTTGGGACTG |
| **Grb10** | **S:** GAAGCAGTACAACGCCCCTA  AS: GTCCTGGTTTGCTCGTCCTC |
| **S12** | S: TGCTGGAGGTGTAATGGACG  AS: CAAGCACACAAAGATGGGCT |

**Table S3.** **miRNAs differentially expressed in GSCs compared to NSCs.**

|  | Table 3A. Upregulated miRNAs in GSCs | | |
| --- | --- | --- | --- |
|  | Mature ID | Fold change GSCs vs NSCs | p value |
| 1 | miR-138 | 94.76 | 0.000 |
| 2 | miR-182 | 78.38 | 0.062 |
| 3 | miR-183 | 20.00 | 0.049 |
| 4 | miR-21 | 8.53 | 0.005 |
| 5 | miR-487b | 8.35 | 0.004 |
| 6 | miR-409-5p | 8.28 | 0.007 |
| 7 | miR-138-1-star | 6.95 | 0.000 |
| 8 | miR-196a | 6.81 | 0.021 |
| 9 | miR-432 | 6.76 | 0.007 |
| 10 | miR-299-3p | 6.27 | 0.009 |
| 11 | miR-409-3p | 6.16 | 0.014 |
| 12 | miR-431 | 5.69 | 0.024 |
| 13 | miR-487a | 5.62 | 0.008 |
| 14 | miR-433 | 5.45 | 0.012 |
| 15 | miR-379 | 5.23 | 0.034 |
| 16 | miR-382 | 5.07 | 0.037 |
| 17 | miR-23a-star | 5.02 | 0.016 |
| 18 | miR-337-5p | 4.92 | 0.015 |
| 19 | miR-126 | 4.45 | 0.048 |
| 20 | miR-24-2-star | 4.42 | 0.044 |
| 21 | miR-154 | 4.37 | 0.018 |
| 22 | miR-376c | 4.32 | 0.022 |
| 23 | miR-134 | 4.31 | 0.042 |
| 24 | miR-21-star | 4.23 | 0.015 |
| 25 | miR-494 | 4.00 | 0.003 |
| 26 | miR-193a-5p | 3.99 | 0.005 |
| 27 | miR-543 | 3.96 | 0.011 |
| 28 | miR-127-3p | 3.91 | 0.030 |
| 29 | miR-381 | 3.69 | 0.050 |
| 30 | miR-654-3p | 3.65 | 0.018 |
| 31 | miR-193b-star | 3.53 | 0.010 |
| 32 | miR-584 | 3.35 | 0.019 |
| 33 | miR-411 | 3.32 | 0.046 |
| 34 | miR-214 | 3.25 | 0.031 |
| 35 | miR-29a | 3.23 | 0.000 |
| 36 | miR-127-5p | 3.21 | 0.032 |
| 37 | miR-671-5p | 3.21 | 0.027 |
| 38 | miR-25-star | 3.02 | 0.016 |
| 39 | miR-485-3p | 3.01 | 0.014 |
| 40 | miR-140-3p | 2.98 | 0.046 |
| 41 | miR-335 | 2.97 | 0.016 |
| 42 | miR-148a | 2.96 | 0.012 |
| 43 | miR-493 | 2.96 | 0.025 |
| 44 | miR-758 | 2.96 | 0.009 |
| 45 | miR-27a-star | 2.69 | 0.006 |
| 46 | miR-654-5p | 2.64 | 0.025 |
| 47 | miR-10b | 2.63 | 0.002 |
| 48 | miR-10b-star | 2.53 | 0.020 |
| 49 | miR-377-star | 2.48 | 0.019 |
| 50 | miR-27a | 2.46 | 0.028 |
| 51 | miR-23b-star | 2.42 | 0.011 |
| 52 | miR-199a-5p | 2.41 | 0.033 |
| 53 | miR-3065-5p | 2.39 | 0.010 |
| 54 | miR-342-5p | 2.38 | 0.011 |
| 55 | miR-431-star | 2.28 | 0.031 |
| 56 | miR-27b-star | 2.14 | 0.054 |
| 57 | miR-339-5p | 2.13 | 0.001 |
| 58 | miR-193b | 2.07 | 0.020 |
| 59 | miR-606 | 2.06 | 0.019 |
| 60 | miR-23a | 1.88 | 0.028 |
| 61 | miR-451 | 1.80 | 0.003 |
| 62 | miR-1306 | 1.72 | 0.040 |
| 63 | miR-3133 | 1.71 | 0.013 |
| 64 | miR-125a-3p | 1.71 | 0.009 |
| 65 | miR-99b-star | 1.70 | 0.043 |
| 66 | miR-492 | 1.64 | 0.003 |
| 67 | miR-154-star | 1.59 | 0.027 |
| 68 | miR-595 | 1.57 | 0.003 |
| 69 | miR-600 | 1.55 | 0.033 |
| 70 | miR-425 | 1.55 | 0.006 |
| 71 | miR-24 | 1.53 | 0.018 |
| 72 | miR-661 | 1.52 | 0.002 |
| 73 | miR-548b-5p | 1.51 | 0.050 |
| 74 | miR-100 | 1.49 | 0.011 |
| 75 | miR-148a-star | 1.48 | 0.046 |
| 76 | miR-98 | 1.47 | 0.011 |
| 77 | miR-3149 | 1.46 | 0.024 |

|  | **Table 3B. Downregulated miRNAs in GSCs** | | |
| --- | --- | --- | --- |
|  | Mature ID | Fold change GSCs vs NSCs | p value |
| **1** | miR-504 | -5.94 | 0.029 |
| **2** | miR-30a-star | -5.22 | 0.017 |
| **3** | miR-891a | -3.91 | 0.006 |
| **4** | miR-9 | -3.38 | 0.000 |
| **5** | miR-30a | -2.96 | 0.000 |
| **6** | miR-9-star | -2.91 | 0.012 |
| **7** | miR-30c-2-star | -2.87 | 0.000 |
| **8** | miR-874 | -2.55 | 0.031 |
| **9** | miR-1287 | -2.50 | 0.019 |
| **10** | miR-532-3p | -2.36 | 0.002 |
| **11** | miR-362-5p | -2.29 | 0.000 |
| **12** | miR-181a-2-star | -2.22 | 0.025 |
| **13** | miR-491-5p | -2.18 | 0.042 |
| **14** | miR-1208 | -2.00 | 0.024 |
| **15** | miR-330-3p | -1.98 | 0.025 |
| **16** | miR-374b | -1.94 | 0.000 |
| **17** | miR-769-5p | -1.87 | 0.004 |
| **18** | miR-501-3p | -1.86 | 0.022 |
| **19** | miR-128 | -1.83 | 0.029 |
| **20** | miR-149 | -1.82 | 0.001 |
| **21** | miR-505-star | -1.66 | 0.004 |
| **22** | miR-660 | -1.58 | 0.019 |
| **23** | miR-1275 | -1.56 | 0.038 |
| **24** | miR-20a | -1.54 | 0.003 |
| **25** | miR-106a | -1.50 | 0.001 |
| **26** | miR-636 | -1.49 | 0.024 |

miRNA lists significantly altered in GSCs compared to NSCs. Fold change>=1.5, p<=0.05.
